# Sjögren’s syndrome-associated microRNAs in CD14^+^ monocytes unveils targeted TGFβ signaling

**DOI:** 10.1186/s13075-016-0987-0

**Published:** 2016-05-03

**Authors:** Adrienne E. G. Williams, Kevin Choi, Annie L. Chan, Yun Jong Lee, Westley H. Reeves, Michael R. Bubb, Carol M. Stewart, Seunghee Cha

**Affiliations:** Departments of Oral and Maxillofacial Diagnostic Sciences, University of Florida College of Dentistry, P.O. Box 100414, Gainesville, FL 32610 USA; Department of Internal Medicine, Seoul National University Bundang Hospital, Seoul, South Korea; Department of Rheumatology and Clinical Immunology, University of Florida College of Medicine, Gainesville, FL 32610 USA

**Keywords:** MicroRNA, Sjögren’s syndrome, Monocytes, TGFβ

## Abstract

**Background:**

Sjögren’s syndrome (SjS) monocytes have a pro-inflammatory phenotype, which may influence SjS pathogenesis. MicroRNAs (miRNAs) are small endogenously expressed molecules that can inhibit protein expression of their targeted genes and have important functions in regulating cell signaling responses. We profiled miRNAs in SjS monocytes to identify a SjS-specific miRNA profile and determine the potential roles of miRNAs in SjS pathogenesis.

**Methods:**

Total RNA was extracted from healthy control (HC, *n* = 10), SjS (*n* = 18), systemic lupus erythematosus (SLE, *n* = 10), and rheumatoid arthritis (RA, *n* = 10) peripheral blood CD14^+^ monocytes for miRNA microarray analysis. To validate select miRNAs from the microarray analysis, the original cohort and a new cohort of monocyte RNA samples from HC (*n* = 9), SjS (*n* = 12), SLE (*n* = 8), and RA (*n* = 9) patients were evaluated by quantitative reverse transcription (RT)-PCR. Functional predictions of differentially expressed miRNAs were determined through miRNA target prediction database analyses. Statistical analyses performed included one-way analysis of variance with Bonferroni post tests, linear regression, and receiver operating characteristic curve analyses.

**Results:**

MiRNAs were predominantly upregulated in SjS monocytes in comparison with controls. Quantitative RT-PCR confirmations supported co-regulation of miR-34b-3p, miR-4701-5p, miR-609, miR-300, miR-3162-3p, and miR-877-3p in SjS monocytes (13/30, 43.3 %) in comparison with SLE (1/17, 5.8 %) and RA (1/18, 5.6 %). MiRNA-target pathway predictions identified SjS-associated miRNAs appear to preferentially target the canonical TGFβ signaling pathway as opposed to pro-inflammatory interleukin-12 and Toll-like receptor/NFkB pathways.

**Conclusions:**

Our results underscore a novel underlying molecular mechanism where SjS-associated miRNAs may collectively suppress TGFβ signaling as opposed to pro-inflammatory interleukin-12 and Toll-like receptor/NFκB pathways in SjS pathogenesis.

**Electronic supplementary material:**

The online version of this article (doi:10.1186/s13075-016-0987-0) contains supplementary material, which is available to authorized users.

## Background

Sjögren’s syndrome (SjS) is a chronic autoimmune disorder that affects the exocrine glands and multiple organ systems. The pathobiology of SjS involves the activation of the innate immune system and development of autoimmunity to exocrine tissues, resulting in severe dryness. In general, SjS can be divided into primary or secondary SjS (pSjS or sSjS) depending on co-morbid rheumatologic or autoimmune diseases [1]. In addition, although SjS is the second most common autoimmune condition to be diagnosed next to rheumatoid arthritis (RA), SjS diagnosis usually is difficult due to multiple symptoms resembling other conditions and a lack of disease-specific diagnostic markers [2, 3]. Devastating consequences of SjS have prompted research endeavors to expedite SjS diagnosis and guide SjS intervention strategies.

The precise mechanisms and sequence of events leading to salivary and ocular dysfunction are still unclear. Inflammatory responses in autoimmune conditions are assumed to be limited based on the tissue microenvironment, where tissue homeostatic processes and regulation of inflammation are critical in curtailing chronic inflammation. The innate immune cells involved in these processes, such as macrophages, dendritic cells (DCs), and epithelial cells, have been shown to shape lymphocyte proliferation and functions during adaptive immune regulation [4]. Monocytes and their derivatives of macrophages and DCs have abnormalities in autoimmune diseases such as systemic lupus erythematosus (SLE), RA, and SjS [5]. Monocytes represent a relatively homogenous population of important precursor cells to peripheral macrophages and DCs. Monocytes have innate immune functions including immunomodulatory functions, cytokine production, and phagocytosis [6]. Among SjS patient cell subsets, monocytes secrete increased levels of pro-inflammatory cytokines such as interleukin(IL)-6 and B cell-activating factor (BAFF) upon stimulation [7], express type I interferon (IFN)-regulated genes [5, 8–10], reduced NFκB inhibitor (IκBα) [11], and show decreased function in phagocytosis of apoptotic cells [12]. Thus, monocytes reflect the inflammatory state in SjS patients [10] and mature monocytes are proposed to contribute to salivary gland inflammation in SjS [13].

MicroRNAs (miRNAs) are naturally occurring post-transcriptional regulators of their targeted genes and can directly inhibit protein translation by a variety of mechanisms including direct cleavage of messenger RNAs (mRNAs) and translational repression due to disruption of translation initiation or premature translation termination [14, 15]. MiRNAs can regulate a wide range of processes including pro-apoptotic pathways, sensitization of innate immune receptor signaling, and cytokine expression in a variety of autoimmune processes and disease [16, 17]. One of the most intriguing aspects is how miRNAs function to cooperatively modulate cell processes by limiting specific pathway components, and thus in recent years extensive research has been performed to characterize miRNAs and their regulation of immune responses and immune cell development. Ours and other studies reported miRNAs are associated with SjS salivary gland tissue inflammation [18–21] and are shown to be upregulated in SjS peripheral blood mononuclear cells [20–23] and in long-term cultured salivary gland-derived epithelial cells [21]. Due to the complexity of cell subsets and miRNA-mRNA interactions involved in autoimmunity, it is still not well understood how miRNA dysregulation contributes to autoimmune disease pathogenesis in human patients.

Considering possible roles of miRNAs as mediators of inflammation, we hypothesized profiling of miRNAs in SjS monocytes provides insight into functional implications of miRNAs in shaping the observed dysregularities. No miRNA studies have been conducted on SjS monocytes to date, let alone clarified implications of miRNAs in SjS pathogenesis. Therefore, the objective of this study was to profile miRNAs in SjS monocytes, focusing on identifying SjS-specific miRNAs and predicting their potential roles in SjS pathogenesis. Our study is the first to characterize the differentially expressed miRNAs in SjS in comparison to HC, SLE, and RA autoimmune conditions and identify a potential SjS-specific miRNA profile in SjS monocytes. Elucidation of the pathobiology of SjS is critical not only to guide intervention strategies, but also to expedite SjS diagnosis.

## Methods

### Patient and control participants

The University of Florida Institutional Review Board approved this study and written permission was obtained from all who participated. Peripheral blood CD14^+^ monocytes were isolated using CD14^+^ whole blood isolation microbeads (Miltenyi Biotec, Bergisch Gladbach, Germany) for miRNA microarray and validation experiments. Patients were diagnosed with SjS according to the 2002 modified European-American diagnostic criteria [1], pSjS (*n* = 21) and sSjS ( *n* = 9) patients were included. Patients with pSjS were recruited when no other rheumatic or underlying disease was identified and sSjS participants were patients diagnosed with rheumatic or additional autoimmune disease. Autoimmune control participants diagnosed with SLE (*n* = 17) or RA (*n* = 18) alone were diagnosed based on the American College of Rheumatology criteria [24, 25]. Healthy donors with no history of autoimmune diseases were included as healthy controls (HC, *n* = 17). Detailed patients’ demographic, clinical and laboratory characteristics are summarized in Additional file 1.

### RNA extraction and sample preparation

Total RNA including small RNAs from frozen CD14^+^ peripheral blood monocytes were extracted with mirVana miRNA Isolation kit (Invitrogen, Carlsbad, CA, USA) according to the manufacturer’s instructions. All RNA sample preparations’ RNA yield and A260/280 ratios were quantified using a NanoDrop1000 spectrophotometer (NanoDrop Technologies, Wilmington, DE, USA) and RNA integrity was assessed by 2100 Bioanalyzer (Agilent Technologies, Santa Clara, CA, USA) prior to microarray profiling and stored at -80 °C until use.

### MiRNA microarray analysis

Microarray miRNA expression profiling experiments were performed for each sample using one-color hybridizations on μParaflo™ microfluidic biochip miRNA microarray platform (LC Sciences, Houston, TX, USA) through LC Sciences’ miRNA microarray service. The array contained 2019 sequence-specific probes covering mature human miRNAs (hsa-) listed in Sanger miRBase Release v.19.0 and 44 Epstein-Barr virus miRNA-specific probes. Hybridized microarrays were scanned using an Axon GenePix 4000B Microarray Scanner and images digitized using Array-Pro Analyzer (MediaCybernetics, Rockville, MD, USA). Microarray data files were subjected background subtraction and to robust LOWESS (locally weighted regression) multi-array average background correction normalization. Data adjustment included signal significance analysis and data filtering that removed miRNAs with undetectable intensity values in more than half of control samples (Additional file 2). The log2-fold change values were calculated for each miRNA between any two participant groups. The threshold value used to define upregulation or downregulation of miRNA was a log2 ratio > ± 0.5 in expression in comparison to HC group and a value of *P* < 0.05 by one-way analysis of variance (ANOVA) with Bonferroni post tests (log2-transformed intensity values) among participant groups.

### Verification of miRNA expression by quantitative reverse transcription (qRT)-PCR

Based on highest ranked increase in expression for SjS in comparison to HC, RA, or SLE controls, six selected candidate miRNAs were verified by qRT-PCR to validate the microarray results and in a separate validation cohort (Additional file 3). MiRNA quantification was carried out applying two-step reaction using commercially available TaqMan miRNA assays (Applied Biosystems, Foster City, CA, USA) according to manufacturer’s protocols. Microarray control samples HC#3727 and #3839, RA#3933, and SLE#3205 were excluded from validation by RT-PCR due to insufficient sample volume. For relative quantification of miRNA expression levels, all samples were normalized against endogenous reference RNA RNU6b using the comparative delta delta cycle threshold (ΔΔCt) method (relative expression level (fold change) = 2^-(∆Ctsample-∆Ctcontrol)^). To minimize experimental variation, matched samples of disease and controls were analyzed on the same reaction plates.

### Verification of gene expression by qRT-PCR

Based on miRNA target prediction three genes *SMAD2*, *SMAD3*, and *SMAD4* were selected for further verification by qRT-PCR in HCs and pSjS monocytes. Gene quantification was carried out using random primers for reverse transcription (high-capacity cDNA reverse transcription kit; Applied Biosystems) and TaqMan gene assay PCR primers (Applied Biosystems) according to manufacturer’s protocols. For relative quantification of gene expression, all samples were normalized against endogenous reference 18S ribosomal RNA using the comparative ΔΔCt method. Values were log2-transformed to display uniform scale for up- and downregulated genes.

### Data analysis and statistical methods

Sample size determinations were performed prior to microarray analyses using software PS:Power and Sample Size Calculation version 3.1.2 [26]. With 80 % power to detect a mean effect of 0.50 with two-sided adjusted type I error probability α = 0.008333 and assuming within-group standard deviation estimate of 0.30 [26], required a sample size of ten subjects per group corresponding to HC, SjS, SLE, and RA. One-way ANOVA followed by Bonferroni post tests were performed as two-sided and adjusted *P* < 0.05 was considered significant (GraphPad Prism v.5 software, GraphPad Software, Inc, La Jolla, CA, USA). Receiver operator characteristics (ROC) curve and binary logistic regression analyses were used to determine the potential miRNA expression value cutoff to discriminate between HC and SjS patients (Additional file 4). Clustering analysis of miRNA microarray signal values was performed using the unsupervised hierarchical clustering method with the Cluster 3.0 program [27] and a clustering plot generated using TreeView software v. 1.6 [28]. MiRNA target prediction was analyzed through use of a variety of databases including validated miRNA targets (DIANA-TarBase v.7.0 [29] and miRSystem program [30]) and precomputed predicted miRNA targets (microRNA.org 2010 release (miRANDA) [31], TargetScan 6.2, RNA22 (miRANDA) [32]), summarized in Additional file 5. Potential biological functions for selected miRNAs were identified by miRSystem program [30]. Hsa-miR-588 has identical seed sequence (position 1–8) with miR-4701-5p and was utilized when database information was not available. Kyoto Encyclopedia of Genes and Genomes (KEGG) program (www.genome.jp/kegg/) was used to map predictions of individual mRNA-miRNA interactions. In addition, previously predicted and validated alternative polyadenylation sites in genes were indicated using NCBI AceView database for human genes [33] and RNAhybrid program (bibiserv.techfak.uni-bielefeld.de/rnahybrid/) was utilized to evaluate predicted miRNA and target gene interactions.

## Results

### MiRNAs are differentially upregulated in SjS monocytes

Microarray expression analyses were performed with HC, SjS, SLE, and RA monocyte samples to identify differentially expressed miRNAs among groups (Table 1).

**Table 1:** MicroRNA microarray log2 ratios for differentially expressed miRNAs of interest

MiRNA	SjS/HC	SjS/RA	SjS/SLE	SLE/HC	SLE/RA	RA/HC
hsa-miR-346	1.04^*^	1.22	0.72	0.32	0.50	-0.19
hsa-miR-629-3p	1.01^*^	1.32	0.58	0.43	0.74	-0.31
hsa-miR-138-1-3p	1.01^*^	1.36	0.57	0.44	0.79	-0.35
hsa-miR-324-3p	0.57	0.83^*^	0.46	0.11	0.37	-0.26
hsa-miR-584-3p	0.82	1.23^*^	0.57	0.24	0.66	-0.41
hsa-miR-1234-3p	0.87^*^	1.38^*^	0.56^*^	0.31	0.82	-0.52
^#^hsa-miR-3162-3p	1.34^*^	1.82^*^	1.06^*^	0.28	0.76	-0.47
hsa-miR-4323	0.87^*^	1.09^*^	0.42	0.45	0.68	-0.22
hsa-miR-34c-3p	1.00^*^	1.23	0.37	0.63	0.85	-0.23
^#^hsa-miR-34b-3p	1.19^*^	1.43^*^	0.75	0.45	0.69	-0.24
hsa-miR-1228-3p	0.85^*^	1.26	0.42	0.43	0.84	-0.41
hsa-miR-4254	0.89^*^	1.03	0.73	0.16	0.30	-0.14
hsa-miR-765	0.25	0.92^*^	0.19	0.06	0.72	-0.66
^#^hsa-miR-877-3p	0.73	1.52^*^	0.55	0.18	0.97	-0.79
^#^hsa-miR-4701-5p	1.00	1.60^*^	0.73	0.27	0.87	-0.60
^#^hsa-miR-300	1.14^*^	1.09^*^	0.72	0.42	0.37	0.05
^#^hsa-miR-609	0.98^*^	0.97^*^	0.71	0.28	0.26	0.01
hsa-miR-485-3p	0.71	1.06^*^	0.45	0.27	0.61	-0.35
hsa-miR-466	0.45	0.13	-0.28	0.73^*^	0.41	0.32
hsa-miR-1273 g-3p	-0.22	-0.11	-0.96^*^	0.74	0.85	-0.12
hsa-miR-5100	0.26	-0.35	-0.59	0.84^*^	0.24	0.60^*^
hsa-miR-3591-3p	0.49^*^	0.66	0.15	0.33	0.50	-0.17
hsa-miR-4270	0.47	1.39^*^	-0.05	0.52	1.43	-0.92
hsa-miR-4778-5p	0.57	0.91^*^	-0.05	0.62^*^	0.96^*^	-0.34

*miRNA* microRNA, *SjS* Sjögren’s syndrome, *HC* healthy control, *RA* rheumatoid arthritis, *SLE* systemic lupus erythematosus

^#^miRNAs selected for validation

^*^ *P* < 0.05 one-way ANOVA with Bonferroni post tests

**Fig. 1:**
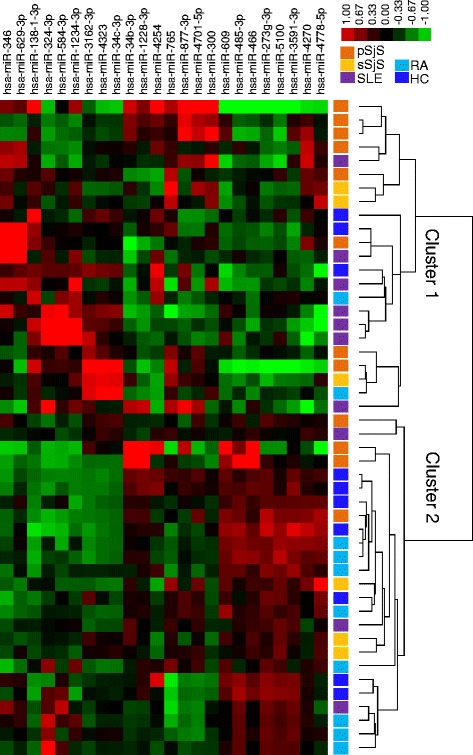
MiRNA expression signature in CD14^+^ monocytes. Microarray heat map showing unsupervised hierarchical clustering analysis of differentially expressed miRNAs (one-way ANOVA with Bonferroni post tests, *P* < 0.05) for SjS (pSjS *n* = 12; sSjS *n* = 6), HC (*n* = 10), SLE (*n* = 10), and RA (*n* = 10) samples. *Green*, low expression levels; *red*, high expression levels. *HC* healthy control, *pSjS* primary Sjögren’s syndrome, *RA* rheumatoid arthritis, *SLE* systemic lupus erythematosus, *SjS* Sjögren’s syndrome, *sSjS* secondary Sjögren’s syndrome

In general, SjS samples showed the highest level of miRNA upregulation, followed by SLE samples with an intermediate level of miRNA expression. Additionally, HC and RA samples showed lower expression levels for these miRNAs. Unsupervised hierarchical clustering (Fig. 1) of the microarray data comparing HC, SjS, SLE and RA CD14^+^ monocytes showed p SjS (8/12, 66.7 %), s SjS (3/6, 50 %), and SLE (7/10, 70 %) samples formed a cluster. Otherwise, most HC (7/10, 70 %) and RA (8/10, 80 %) samples were grouped within a second cluster.

**Fig. 2:**
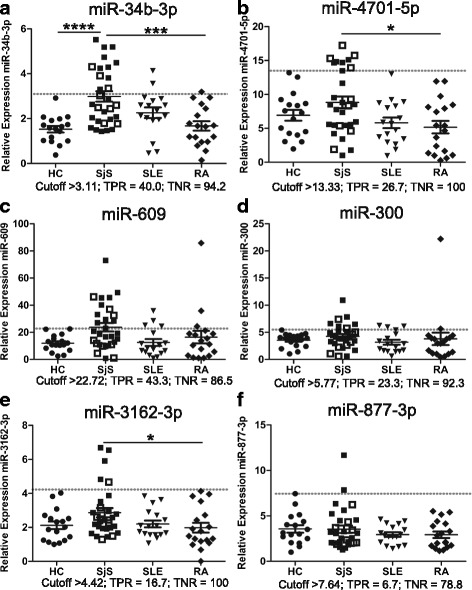
SjS-associated miRNAs are upregulated in CD14^+^ monocytes. Expression verification by qRT-PCR for combined microarray and validation cohorts including HC (*circle*, *n* = 17), SjS (pSjS *closed square*, *n* = 21; sSjS *open square*, *n* = 9), SLE (*triangle*, *n* = 17), and RA (*diamond*, *n* = 18) samples. *Horizontal lines* and *bars* represent mean ± SEM. *Dotted line* indicates cutoff value determined by ROC curve analyses (Additional file 4: Figure S2). True positive rate (TPR, sensitivity) = true positive/(true positive + false negative), true negative rate (TNR, specificity) = true negative/(true negative + false positive). ^*^ *P* < 0.05, ^***^ *P* < 0.001, ^****^ *P* < 0.0001 by one-way ANOVA with Bonferroni post tests. *HC* healthy control, *pSjS* primary Sjögren’s syndrome, *RA* rheumatoid arthritis, *SLE* systemic lupus erythematosus, *SjS* Sjögren’s syndrome, *sSjS* secondary Sjögren’s syndrome

We next used qRT-PCR to verify our microarray findings for selected miRNAs. These six miRNAs included miR-34b-3p, miR-300, miR-609, miR-877-3p, miR-3162-3p, and miR-4701-5p. The expression levels of these miRNAs from the combined cohort of samples are presented in Fig. 2. Of note, miR-34b-3p displayed a significant differential expression between SjS and HC or RA from the combined cohort (Fig. 2a). In addition, miR-4701-5p, and miR-3162-3p were significantly upregulated in SjS patient samples with comparison to RA (Fig. 2b,e). Although miR-609 and miR-300 tended to be upregulated in SjS compared with other control groups (Fig. 2c,d), there were no significant differences between relative expressions. Furthermore, ROC curve analyses were performed to determine cutoff values that maximized the specificity of discrimination between HC and SjS samples (Fig. 2, Additional file 4).

### SjS-associated monocyte miRNAs are co-expressed

**Fig. 3:**
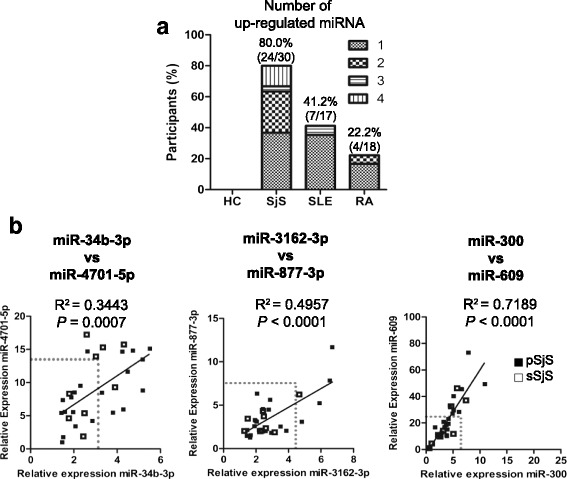
SjS-associated miRNA expression levels are positively associated in CD14^+^ monocytes. **a** Number and percent distribution of upregulated miRNAs in SjS (*n* = 30), SLE (*n* = 17), and RA (*n* = 18) patients. **b** Linear regression analyses were used to define associations between miRNA expression levels in SjS patients (pSjS *closed square*, *n* = 21; sSjS *open square*, *n* = 9). Cutoff values determined by ROC curve analyses are indicated by *dotted box. P* < 0.05 was considered statistically significant. *HC* healthy control, *miRNA* microRNA, *RA* rheumatoid arthritis, *SLE* systemic lupus erythematosus, *SjS* Sjögren’s syndrome

MiRNAs are unlikely to be unique to a particular disease process and co-regulation may indicate similar functions. Therefore, we pursued characterization of their respective co-expression to determine whether a predictive panel could be identified. Utilizing cutoff values reported in Fig. 2 for our six selected miRNA markers, we evaluated the number of upregulated miRNAs in our combined cohort (Fig. 3a). Of note, 43.3 % of SjS patients (13/30) had upregulated expression of at least two of the six SjS-associated miRNAs. In contrast, 5.8 % SLE (1/17) and 5.6 % RA (1/18) had at least two upregulated SjS-associated miRNAs (Fig. 3a).

Since differentially expressed miRNAs tended to be co-increased in SjS monocytes, we tested whether any pairs of miRNAs displayed significant relationships in their expression levels. Linear regression analyses indicated miR-34b-3p and miR-4701-5p; miR-609 and miR-300; and miR-3162-3p and miR-877-3p expression levels were positively associated in SjS patients (Fig. 3b). Additionally, miR-609 and miR-300 expression values were positively associated in HC, SLE, and RA samples and miR-34b-3p and miR-4701-5p were also associated in RA patients (Additional file 6).

### SjS-associated monocyte miRNAs preferentially target TGFβ signaling pathways

**Fig. 4:**
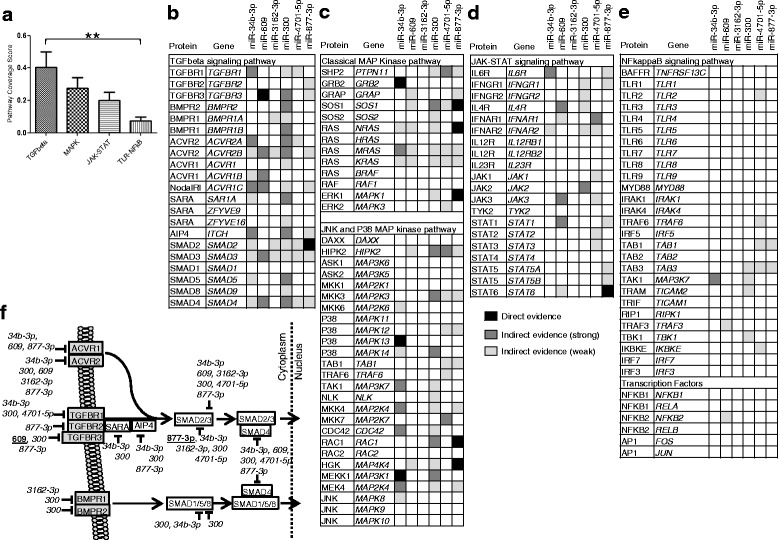
SjS-associated miRNAs are predicted to target canonical TGFβ signaling network. **a** Summary of average pathway-coverage scores for the six SjS-associated miRNAs for TGFβ, MAPK, JAK-STAT, and TLR-NFκB signal transduction pathways. *Bars* represent mean percent coverage ± SD for the six miRNAs. ^**^ *P* < 0.01 by one-way ANOVA with Bonferroni post tests. MiRNA target predictions for TGFβ (**b**), MAPK (**c**), JAK-STAT (**d**), and TLR-NFκB (**e**) were analyzed through databases for validated miRNA targets (*black*, direct evidence) and precomputed predicted miRNA targets (*light gray*, indirect evidence (weak) = 2 programs and *dark gray*, indirect evidence (strong) = 3 or more programs). **f** Visualization of predicted miRNA targeting of canonical TGFβ signal transduction pathway

High-throughput miRNA target validation strategies are currently limited. Therefore, multiple miRNA target prediction algorithms and validated miRNA-target interaction databases were incorporated to identify potential functions of SjS-associated miRNAs in monocytes (summarized in Additional file 5). MiRNA target prediction was analyzed through databases for validated miRNA targets (direct evidence) and precomputed predicted miRNA targets (indirect evidence (weak) = 2 programs and indirect evidence (strong) = 3 or more programs). Our analyses further incorporated the KEGG program to map potential miRNA-regulated pathways. The canonical transforming growth factor beta (TGFβ) signaling pathway showed an average pathway coverage of 40.5 % ± 23.1 (SD) for the six SjS-associated miRNAs (Fig. 4a). Key factors such as *TGFBR3* and *SMAD2* involved in TGFβ signaling have direct evidence for targeting by miR-609 and miR-877-3p, respectively (Fig. 4b).

Additionally, MAPK pathways, which are also activated through TGFβ signaling receptors [34], also tended to show potential regulation (27.4 % ± 16.4 pathway coverage) by SjS-associated miRNAs (Fig. 4a). MiR-34b-3p has direct evidence for targeting *GRB2*, P38/*MAPK13*, and MEKK1/*MAP3K1* and miR-877-3p has direct evidence for targeting *SOS1*, *NRAS*, ERK1/*MAPK1*, *RAC1*, and HGK/*MAP4K4* (Fig. 4c). Visualization of the MAPK signaling pathways indicated critical components of the classical MAPK, JNK, and p38 MAPK signaling pathways may be affected by SjS-associated miRNAs (Additional file 7A).

Furthermore, evaluation of JAK-STAT signaling cascades, which are important for cytokine signaling cascades, also showed potential regulation (20.0 % ± 12.2 pathway coverage) by SjS-associated miRNAs (Fig. 4a). Of note, miR-877-3p had direct evidence for targeting *STAT6* (Fig. 4d), which codes for the IL-4/IL-13 pathway transcription factor. In contrast, none of the SjS-associated miRNAs were predicted to target IL-12 receptor (*IL12RB1*, *IL12RB2*), *TYK2*, or *STAT4* (Fig. 4d), which are involved in the IL-12 signaling cascade (Additional file 7B). Furthermore, the pro-inflammatory TLR/NFκB cascades averaged 4.2 % ± 3.2 pathway coverage (Fig. 4a,e) and had no direct evidence for targeting by SjS-associated miRNAs.

**Fig. 5:**
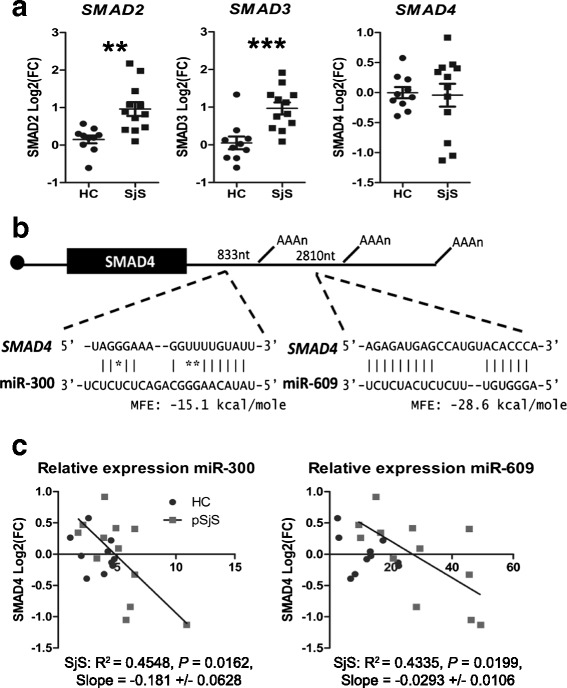
MiR-300 and miR-609 are associated with inhibition of SMAD4 gene expression. **a** Relative expression of *SMAD2*, *SMAD3*, and *SMAD4* were evaluated by qRT-PCR for SjS (*n* = 12) and HC (*n* = 10) CD14^+^ monocytes. Data represents log2-transformed gene expression. ^**^ *P* < 0.01, ^***^ *P* < 0.001 by two-tailed *t* test. **b** Target prediction of *SMAD4* 3′UTR by miR-300 and miR-609 represents target-miRNA alignments with the highest predicted minimum free energy (MFE). Position of alternative polyadenylation sites are indicated in the *SMAD4* 3′UTR. **c** Linear regression analyses of miR-300 and miR-609 with *SMAD4* gene expression. *HC* healthy control, *pSjS* primary Sjögren’s syndrome, *SjS* Sjögren’s syndrome

To identify impact of SjS-associated miRNAs on TGFβ signaling molecule gene levels in CD14+ monocytes, relative gene expression of *SMAD2*, *SMAD3*, and *SMAD4* were evaluated by qRT-PCR. *SMAD2* and *SMAD3* gene expression levels were significantly increased in pSjS monocytes compared with HCs, although *SMAD4* gene expression tended to be reduced (Fig. 5a). Further sequence analyses of predicted miRNA target sites revealed multiple alternative polyadenylation sites upstream of the predicted miRNA binding sites in the *SMAD2* and *SMAD3* 3′ untranslated region (UTR) [33]. In contrast, analyses of *SMAD4* 3′UTR indicated miR-300 could possibly interact at four sites (positions 745, 833, 1094, 1185 3’UTR) upstream of the first alternative polyadenylation site (position 1499 [33]) and miR-609 to interact downstream (position 2810) in the *SMAD4* 3’UTR (Fig. 5b). Regression analyses indicated significant associations for both miR-300 and miR-609 with reductions in *SMAD4* gene expression in pSjS patient monocytes (Fig. 5c).

## Discussion

Research on gene expression in SjS CD14^+^ monocytes has yielded valuable findings for the role of inflammatory genes in SjS [5]. However, identifying master genes to be targeted to reverse disease processes has been a challenge in the field of SjS. MiRNAs can modulate protein expression levels by inhibiting gene translation into protein. MiRNA expression dysregulation has been reported in a variety of autoimmune diseases, including SjS [18–20, 22, 23]. However, the vast majority of previous studies evaluated miRNA expression levels in mixed cell populations or tissues, which may confound the further evaluation of specific miRNA-mRNA target relationships. Therefore, our study utilized miRNA microarray analyses to screen for differentially expressed miRNAs in SjS CD14^+^ monocytes in comparison with HC, RA, and SLE. In addition, we selected six SjS-associated miRNAs to be validated by RT-PCR and performed intensive pathway analyses to identify potential target pathways of SjS-associated miRNAs in CD14^+^ monocytes.

In general, primary and secondary SjS patients were undifferentiated from each other and showed the highest level of miRNA expression, whereas SLE patients tended to have an intermediate level of expression in comparison to SjS patients. Unsupervised hierarchical clustering of these miRNAs showed SjS and SLE patients clustered together independent from HCs and RA patient miRNA profiles (Fig. 1). These findings indicate miRNA alterations in monocytes are more common in SjS regardless of co-morbid autoimmune conditions and their dysregulation may play a role in SjS and SLE. In addition, although the healthy control group tended to be much younger than autoimmune groups (Additional file 1), they did not differ significantly based on miRNA associations with age (Additional file 8), which suggests age is not likely to confound our results.

It is important to note the monocytes utilized in this study were freshly isolated from peripheral blood. Monocytes once activated will rapidly adhere and migrate into affected tissues. Therefore, it was unsurprising a variety of previously reported miRNAs associated with monocyte activation and differentiation into DCs and macrophages, including miR-155-5p, miR-146a-5p, miR-146b-5p, miR-21-5p, miR-22-3p, miR-221-3p, miR-222-3p, and miR-424-5p [35, 36] were not differentially expressed miRNAs between SjS and control groups in our study. Indicating upregulated SjS-associated miRNAs in monocytes are most likely independent of immune activation stimuli controlling monocyte maturation and differentiation.

Validation of six selected SjS-associated miRNAs showed miR-34b-3p was upregulated in SjS patient monocytes in comparison to HCs and RA patient monocytes (Fig. 2a). MiRNAs significantly upregulated in SjS patient monocytes in comparison to RA patients were miR-3162-3p and miR-4701-5p (Fig. 2b,e). Notably, miR-300 and miR-609 tended to be elevated in SjS samples, although two RA patients from the validation cohort expressed higher levels of miR-300 and miR-609 (Fig. 2c,d). Conceivably, this observed overlap in upregulated SjS-associated miRNAs for SLE and RA patients could be related to the observed co-aggregation and similarities in serologic profiles of SjS patients with SLE and RA [37, 38].

Because SjS-associated miRNAs were similarly upregulated in SjS patients, we hypothesized SjS-associated miRNAs may also be co-expressed in individuals. Interestingly, 43.3 % of SjS patients, in comparison to 5.8 % SLE and 5.6 % RA patients, had upregulated expression of at least two of the six SjS-associated miRNAs analyzed (Fig 3a). Furthermore, regression analyses indicated definite pairs of miRNAs showed positive associations in SjS patients (Fig. 3b). Therefore, our data suggests co-expression of SjS-associated miRNAs may be especially useful in distinguishing cases of SjS. In addition, since these SjS-associated miRNAs are not genetically linked (Additional file 5), co-expression and upregulation of these miRNAs in SjS may indicate shared functions of regulating molecular pathways in monocytes.

In general, co-expressed miRNAs can share functions either by co-targeting individual genes or by targeting different components of the same pathway. Our in-depth analyses of target prediction algorithms and verified miRNA-mRNA interactions were utilized to reconstruct potential pathways targeted by SjS-associated miRNAs. Interestingly, canonical TGFβ signaling pathway showed the greatest coverage of predicted targets, averaging 40.5 % ± 23.1 (SD) pathway coverage for the six SjS-associated miRNAs (Fig. 4a). Moreover, TGFβ receptors are well known to activate a variety of associated signal transduction cascades, including the canonical MAP kinase and p 38/JNK MAP kinase pathways [34]. Targeting of the MAPK pathways averaged 27.4 % ± 16.4 coverage for the six SjS-associated miRNAs tested (Fig. 4a). Previously, miR-34 family members [39, 40], miR-609 [40], and miR-300 [41] were independently predicted to target the TGFβ signaling pathway. Specifically, experimental data support miR-34b-3p directly targets cAMP response element-binding (CREB)-1 transcription factor [42] and bone morphogenetic protein (BMP)-2 [43]. In addition, miR-609 directly targeted the full-length gene transcript of TGFβ1 [44] and *TGFBR3* gene transcript [29, 45]. MiR-300 overexpression inhibited the TGFβ-mediated epithelial-to-mesenchymal transition in tumor cells [46]. And miR-877-3p has been shown to directly target *SMAD2* gene transcript [29, 47]. Furthermore, we observed considerable overlap in evidence for SjS-associated miRNAs to target *ACVR2B*, *SMAD2*, *SMAD3*, and *SMAD4* (Fig. 4b).

Based on our target prediction analyses, *SMAD2*, *SMAD3* and *SMAD4* were selected for initial screening for changes in gene expression in SjS monocytes compared with HCs. Intriguingly, *SMAD2* and *SMAD3* gene expression indicated significant increases in SjS monocytes compared with HCs (Fig. 5a), which has not been previously reported. Both *SMAD2* and *SMAD3* 3’UTRs contain multiple alternative polyadenylation sites upstream of predicted miRNA binding sites according to AceView gene database [33], which we speculate could result in failure in miRNA-directed inhibition of gene transcript levels. Alternatively, miRNAs can also regulate protein translation by mechanisms other than transcript degradation [15]. However, at this time our study is limited to RNA-based analyses and monocytes are collected analyses. Relationships between miRNAs and their post-transcriptional inhibition of target genes will be addressed as soon as sufficient human monocytes are collected for protein analyses.

Nevertheless, *SMAD4* gene expression exhibited repression in a subpopulation of pSjS patients (Fig. 5a). The SMAD4 protein of the family of TGFβ signaling molecules acts as an essential common mediator for receptor-regulated SMADs to enter the nucleus. MiR-300 and miR-609, which showed the strongest co-association, are likely to target *SMAD4* directly based on target prediction analyses (Fig. 4b). Four miR-300 putative interaction sites are upstream of the first alternative polyadenylation site and the best predicted interaction of miR-300 with the lowest minimum free energy (MFE) -15.1 kcal/mole begins at nucleotide position 833 of the *SMAD4* 3’UTR as is shown in Fig. 4b. The best predicted interaction for miR-609, MFE -28.6 kcal/mole, is relatively nearby downstream to miR-300 predicted target sites and begins at position 2810 of the *SMAD4* 3’UTR (Fig. 4b). Regression analyses showed *SMAD4* gene expression is significantly associated with both miR-300 and miR-609 (Fig. 4c), indicating possible cooperative targeting of *SMAD4*. Although further studies to identify target protein levels are required to define specific targets and functions of SjS-associated miRNAs in monocytes, our results indicate SjS-associated miRNAs could cooperate to modulate gene expression.

Our extensive data analyses pinpointing the TGFβ signaling network prompted us to revisit potential roles of TGFβ signaling in SjS pathogenesis. Until now the involvement of TGFβ in SjS patients has been largely unappreciated due to previous inconsistencies in reported TGFβ protein expression levels in SjS patient lip biopsy tissues [48–52] and in circulation [53, 54]. Conditional knockout of TGFβ receptors in mice have been utilized to better identify roles of TGFβ signaling in autoimmunity. Interestingly, TGFβ receptor 1 (TGFβR1) conditional knockout in mice salivary glands developed glandular inflammation resembling SjS only in female mice [55]. Of interest to our current study, bone marrow-derived DC-specific deletion of TGFβ receptor 2 (TGFβR2) resulted in multi-organ inflammation and activation of autoreactive T and B cells [56]. TGFβR2-deficient DCs are more pro-inflammatory (increased stimulated TNF, IL-6, IL-12, IL-1β, and IFNγ gene expression [56, 57]) and also have impaired IL-4 polarization of M2 alternatively activated regulatory macrophages [58]. Altogether these studies signify intact TGFβ signal transduction is critical for maintaining a regulatory immune phenotype and reinforcing DC/macrophage control of autoimmune progression.

Our target prediction analyses also identified SjS-associated miRNAs were predicted to target members of the JAK-STAT signaling family, averaging 20.0 % ± 16.4 coverage of signaling molecules (Fig. 4a). The MAPK and JAK-STAT signaling pathways are well known to affect a variety of pro-inflammatory and immune-modulatory cascades beyond TGFβ family signaling. As such, we propose that SjS-associated miRNAs also function by limiting the availability of shared signaling molecules, which creates conditions for competition that would ultimately favor pathways with greater abundance of signaling molecules. Substrate competition is found in many types of biological processes and can influence dynamics and steady state concentrations of a pathway [59] as well as serve as a general signal integration strategy for networks where enzymes interact with multiple regulators and substrates [60].

Interestingly, pro-inflammatory IL-12 and IL-23 signaling pathway molecules *IL12RB1*, *IL12RB2*, *IL23R*, *TYK2*, and *STAT4*, were not predicted to be targeted by SjS-associated miRNAs (Fig. 4d). Previously, IL-12p40 secretion in SjS patient monocyte-derived DCs was increased and correlated with increased NFκB/RELB protein levels [61]. Likewise, our analyses of the NFκB signaling pathway showed relatively low level of pathway coverage (7.3 % ± 5.8) by SjS-associated miRNAs (Fig. 4a,e). Intriguingly, SjS patient monocytes are previously shown to have increased gene expression of NFκB transcription factors (*NFKB1* and *NFKB2*) and display increased inflammatory responses to BAFF and IFNγ [7, 9, 61]. Notably, SjS patients’ monocytes also exhibit reduced levels of inhibitor IκBα, which is a central regulator of NFkB activation [11] and TGFβ signaling is critical for IκBα maintenance [62–64]. Therefore, pro-inflammatory IL-12 and NFκB signaling pathways are presumed to be well maintained, whereas SjS-associated miRNAs skew regulatory TGFβ signaling responses in SjS monocytes.

## Conclusions

Our study identified for the first time a miRNA signature for SjS in CD14^+^ monocytes, which appears to preferentially target TGFβ signaling responses as opposed to pro-inflammatory signaling pathways in monocytes. The interplay of the opposing NFκB and TGFβ signaling pathways is essential for coordinated cellular responses and numerous studies have established a critical role for intact TGFβ signaling mechanisms in controlling autoimmunity. As such, our study provides critical insight into the observed increase in inflammatory responses in SjS patient monocytes, which may possibly be related to changes in the salivary gland as well. Furthermore, we propose a paradigm shift in favor of defective regulatory signaling as an underlying mechanism for the observed pro-inflammatory phenotype in SjS monocytes. Clinical associations with miRNAs will need to be evaluated from a larger data set. Further examination of predicted miRNA-targeted protein levels and signaling responses in monocytes and in SjS salivary gland cell subsets is required to define the role of these upregulated miRNAs in SjS pathogenesis.

## Additional files

Additional file 1: Table S1. Demographic and clinical parameters from the microarray, validation, and combined cohorts. (DOCX 25 kb) Additional file 2: Table S2. MicroRNA microarray data. (XLSX 4872 kb) Additional file 3: Figure S1. Differential miRNA expression verification by qRT-PCR from microarray and an independent validation cohort for CD14^+^ monocyte samples. HC, *circles*; pSjS, *closed squares*; sSjS, *open squares*; SLE, *triangle*; RA, *diamond. Dotted line* indicates cutoff value established by ROC curve analyses. ^*^ *P* < 0.05 by one-way ANOVA with Bonferroni post tests. (PDF 64 kb) Additional file 4: Figure S2. SjS-associated miRNA ROC curve analyses summary from qRT-PCR data. ROC curve analyses were performed for indicated miRNAs comparing HC (*n* = 17) and primary SjS (*n* = 21) samples. *P* < 0.05 was considered statistically significant. Cutoff values were determined to maximize specificity. (PDF 118 kb) Additional file 5: Table S3. Database analysis for miRNA target prediction summary. ^†^Databases contain direct miRNA-mRNA target interaction information. (DOC 32 kb) Additional file 6: Figure S3. SjS-associated miRNA expression levels are positively associated in CD14^+^ monocytes. Linear regression analyses were used to define associations between miRNAs in HC (*circle*, *n* = 17), SLE (*triangle*, *n* = 17), and RA (*diamond*, *n* = 18) patient groups. Cutoff values established by ROC curve analyses are indicated by *dotted lines. P* < 0.05 was considered statistically significant. (PDF 125 kb) Additional file 7: Figure S4. MAP-kinase and JAK-STAT signaling components are predicted targets of SjS-associated miRNAs. Visualization of predicted miRNA targeting of the MAPK (A) and JAK-STAT (B) signaling pathways. Specific mRNA-miRNA interaction results were based on direct evidence obtained from DIANA Tarbase V.6 database (*underlined*) and from multiple target prediction programs. (PDF 83 kb) Additional file 8: Figure S5. Analysis of association of age with miRNA expression level indicates no significant differences between HC, SjS, SLE, and RA groups. Based on linear regression analyses, the differences between slopes were not significant among HC, SjS, SLE, and RA groups for miR-34b-3p (*P* = 0.1974), miR-4701-5p (*P* = 0.3065), miR-609 (*P* = 0.511), miR-300 (*P* = 0.1904), miR-3162-3p (*P* = 0.4748), and miR-877-3p (0.9712). In addition, although HCs tended to be younger than autoimmune patients on average, they are within the age range of autoimmune patients. (PDF 68 kb)

## Abbreviations

ANOVA: analysis of variance
BAFF: B cell-activating factor
BMP-2: bone morphogenetic protein 2
CREB1: cAMP response element binding-1
DC: dendritic cell
HC: healthy control
Hsa: *Homo sapiens*
IFN: interferon
IL: interleukin
IκBα: nuclear factor of kappa light polypeptide gene enhancer in B cells inhibitor, alpha
JAK: Janus kinase
KEGG: Kyoto Encyclopedia of Genes and Genomes
LOWESS: locally weighted scatterplot smoothing
MAPK: mitogen-activated protein kinase
MFE: minimum free energy
miRNA: microRNA
mRNA: messenger RNA
NFκB: nuclear factor kappa-light-chain-enhancer of activated B cells
pSjS: primary SjS
RA: rheumatoid arthritis
ROC: receiver operating characteristic
RT: reverse transcription
SjS: Sjögren’s syndrome
SLE: systemic lupus erythematosus
SMAD: Sma and Mad (mothers against decapentaplegic)
sSjS: secondary SjS
STAT: signal transducer and activator of transcription
TGFβ: transforming growth factor beta
TGFβR1: transforming growth factor beta receptor type 1
TGFβR2: transforming growth factor beta receptor type 2
TLR: Toll-like receptor
UTR: untranslated region
ΔΔCt: delta delta cycle threshold
