# Supplementary material for: Sjögren’s syndrome-associated microRNAs in CD14+ monocytes unveils targeted TGFβ signaling
Source: Arthritis Res Ther. 2016 May 3;18:95. doi: 10.1186/s13075-016-0987-0 (PMC4855899; doi:10.1186/s13075-016-0987-0)
Supplement: Additional file 1: Table S1. — Demographic and clinical parameters from the microarray, validation, and combined cohorts. (DOCX 25 kb) [file 13075_2016_987_MOESM1_ESM.docx]

**Table S1** Demographic and clinical parameters from the microarray, validation, and combined cohorts.

| Microarray Cohort | HC | pSjS | sSjS | SLE | RA |
| --- | --- | --- | --- | --- | --- |
| Total no. | 10 | 12 | 6 | 10 | 10 |
| Age, mean ± SD years | 34.5 ± 12.5 | 52.4 ± 14.2 | 46.7 ± 18.3 | 48.0 ± 17.1 | 56.4 ± 16.1 |
| Sex, no. (%) female | 9 (90.0) | 12 (100) | 6 (100) | 10 (100) | 6 (60.0) |
| Secondary diseases, no. | NA | NA | 4 SLE,  1 Raynaud’s,  1 Vasculitis | NA | NA |
| Anti-SSA/Ro+, no. (%) | NA | 10/11 (90.9) | 4/4 (100) | 4/10 (40.0) | NA |
| Anti-SSB/La+, no. (%) | NA | 7/11 (63.6) | 3/3 (100) | 2/10 (20.0) | NA |
| Anti-RF+, no. (%) | NA | 3/3 (100) | 2/2 (100) | NA | 7/10 (70.0) |
| ANA+, no. (%) | NA | 8/8 (100) | NA | 10/10 (100) | NA |
| Focus score+, no. (%) | NA | 4/4 (100) | NA | NA | NA |
| Salivary flow ≤ 0.1 mL/min., no. (%) | NA | 2/7 (28.6) | 0/1 (0.0) | NA | NA |
| Rheumatoid nodules | NA | NA | NA | NA | 2/10 (20.0) |
| Radiographic changes | NA | NA | NA | NA | 4/9 (44.4) |

| Validation Cohort | HC | pSjS | sSjS | SLE | RA |
| --- | --- | --- | --- | --- | --- |
| Total no. | 9 | 9 | 3 | 8 | 9 |
| Age, mean ± SD years | 33 ± 8 | 62.1 ± 12.5 | 68.3 ± 12.5 | 59.5 ± 12.1 | 50.5 ± 17.0 |
| Sex, no. (%) female | 9 (100) | 8 (88.9) | 3 (100) | 7 (87.5) | 6/8 (75.0) |
| Secondary diseases, no. | NA | NA | 2 SLE,  1 PBC | NA | NA |
| Anti-SSA/Ro+, no. (%) | NA | 7/9 (77.8) | 2/3 (66.7) | 2/8 (25.0) | NA |
| Anti-SSB/La+, no. (%) | NA | 2/8 (25.0) | 1/3 (33.3) | 0 (0.0) | NA |
| Anti-RF+, no. (%) | NA | 3/4 (75.0) | 2/2 (100) | NA | 5/8 (62.5) |
| ANA+, no. (%) | NA | 5/5 (100) | 2/2 (100) | 6/8 (75.0) | NA |
| Focus score+, no. (%) | NA | 3/3 (100) | 1/1 (100) | NA | NA |
| Salivary flow ≤ 0.1 mL/min., no. (%) | NA | 3/7 (42.9) | 3/3 (100) | NA | NA |
| Rheumatoid nodules | NA | NA | NA | NA | 0 (0.0) |
| Radiographic changes | NA | NA | NA | NA | 5/8 (62.5) |
| Combined Cohort | HC | pSjS | sSjS | SLE | RA |
| Total no. | 17 | 21 | 9 | 17 | 18 |
| Age, mean ± SD years | 34.2 ± 10.6 | 56.6 ± 14.0 | 53.9 ± 19.0 | 51.3 ± 14.2 | 53.9 ± 16.8 |
| Sex, no. (%) female | 16 (94.1) | 20 (95.2) | 9 (100) | 16 (94.1) | 11/17 (64.7) |
| Secondary diseases, no. | NA | NA | 6 SLE,  1 PBC  1 Vasculitis  1 Raynaud’s | NA | NA |
| Anti-SSA/Ro+, no. (%) | NA | 17/20 (85) | 6/7 (85.7) | 6/17 (35.3) | NA |
| Anti-SSB/La+, no. (%) | NA | 9/19 (47.4) | 4/6 (66.7) | 2/17 (11.8) | NA |
| Anti-RF+, no. (%) | NA | 6/7 (85.7) | 4/4 (100) | NA | 12/17 (70.6) |
| ANA+, no. (%) | NA | 13/13 (100) | 4/4 (100) | 15/17 (88.2) | NA |
| Focus score+, no. (%) | NA | 7/7 (100) | 1/1 (100) | NA | NA |
| Salivary flow ≤ 0.1 mL/min., no. (%) | NA | 5/14 (35.7) | 3/4 (75) | NA | NA |
| Rheumatoid nodules | NA | NA | NA | NA | 1/17 (5.9) |
| Radiographic changes | NA | NA | NA | NA | 8/16 (50) |
