# Supplementary figures and images for: Sjögren’s syndrome-associated microRNAs in CD14+ monocytes unveils targeted TGFβ signaling
Source: Arthritis Res Ther. 2016 May 3;18:95. doi: 10.1186/s13075-016-0987-0 (PMC4855899; doi:10.1186/s13075-016-0987-0)

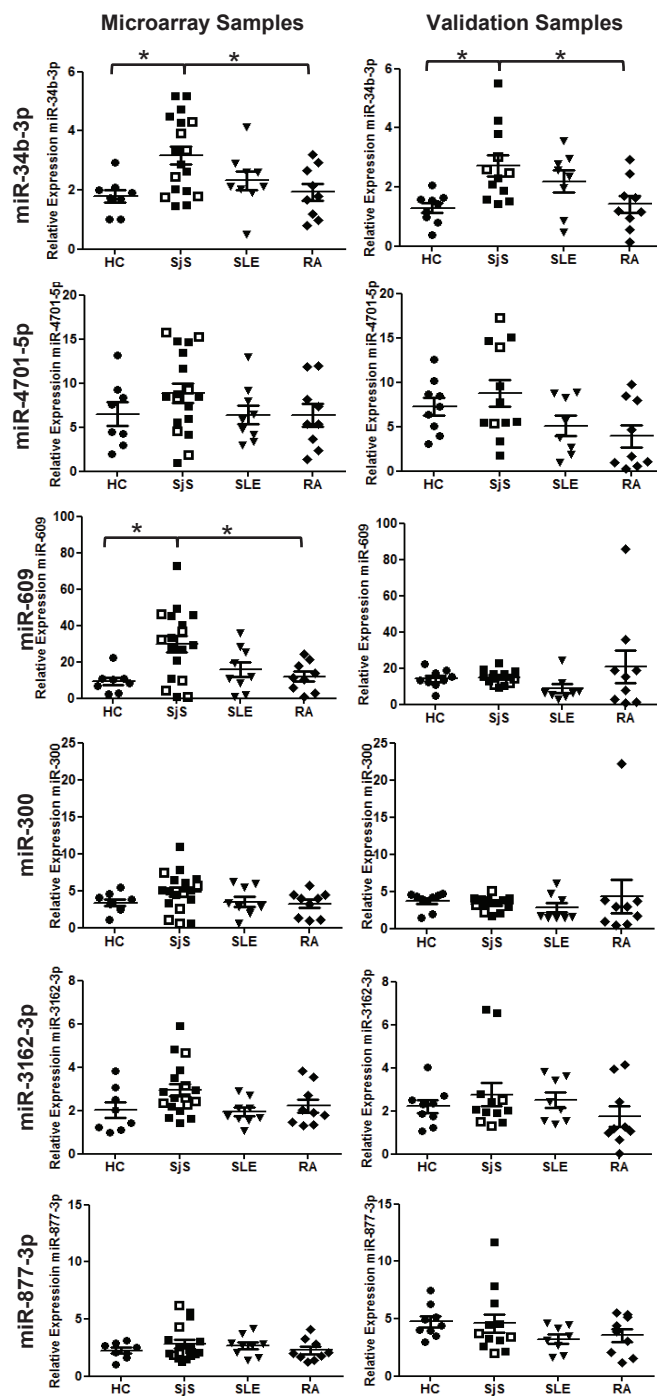

Supplement: Additional file 3: Figure S1. — Differential miRNA expression verification by qRT-PCR from microarray and an independent validation cohort for CD14+ monocyte samples. HC, circles; pSjS, closed squares; sSjS, open squares; SLE, triangle; RA, diamond. Dotted line indicates cutoff value established by ROC curve analyses. * P < 0.05 by one-way ANOVA with Bonferroni post tests. (PDF 64 kb) [file 13075_2016_987_MOESM3_ESM.pdf]

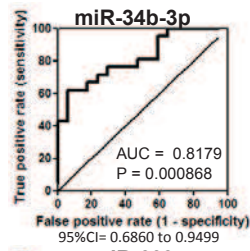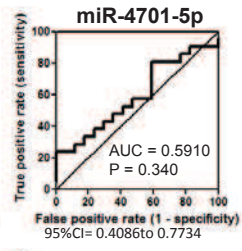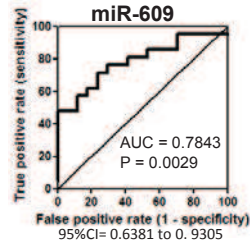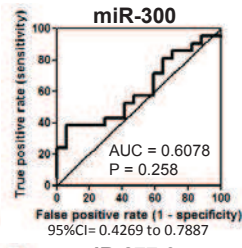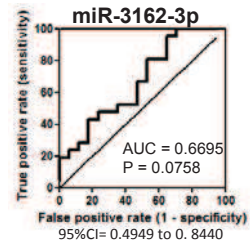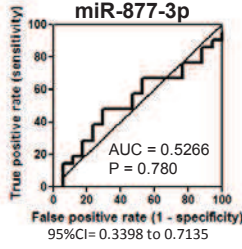

Supplement: Additional file 4: Figure S2. — SjS-associated miRNA ROC curve analyses summary from qRT-PCR data. ROC curve analyses were performed for indicated miRNAs comparing HC (n = 17) and primary SjS (n = 21) samples. P < 0.05 was considered statistically significant. Cutoff values were determined to maximize specificity. (PDF 118 kb) [file 13075_2016_987_MOESM4_ESM.pdf]

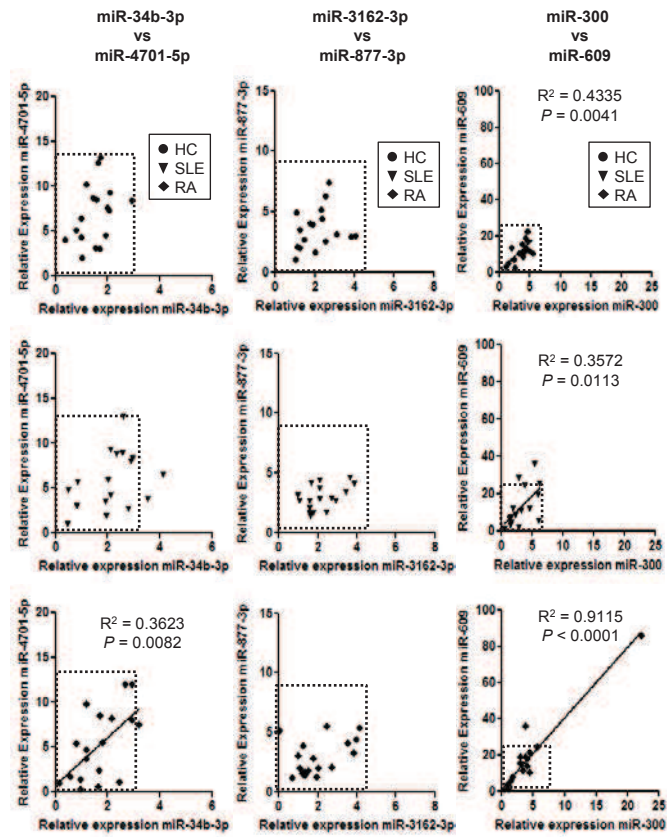

Supplement: Additional file 6: Figure S3. — SjS-associated miRNA expression levels are positively associated in CD14+ monocytes. Linear regression analyses were used to define associations between miRNAs in HC (circle, n = 17), SLE (triangle, n = 17), and RA (diamond, n = 18) patient groups. Cutoff values established by ROC curve analyses are indicated by dotted lines. P < 0.05 was considered statistically significant. (PDF 125 kb) [file 13075_2016_987_MOESM6_ESM.pdf]

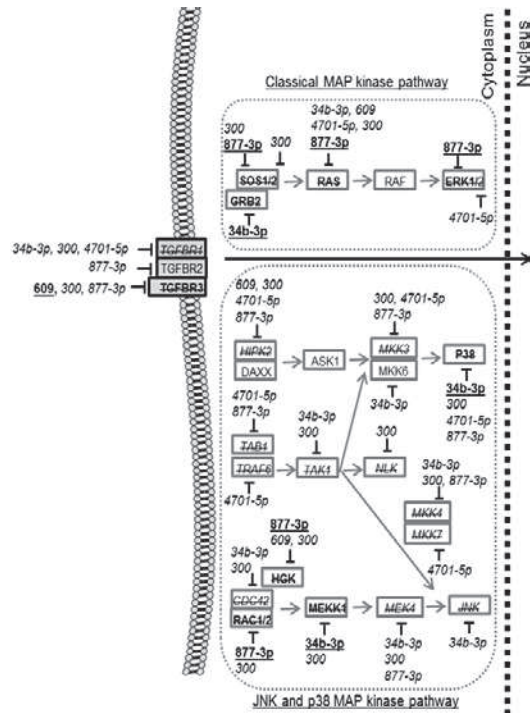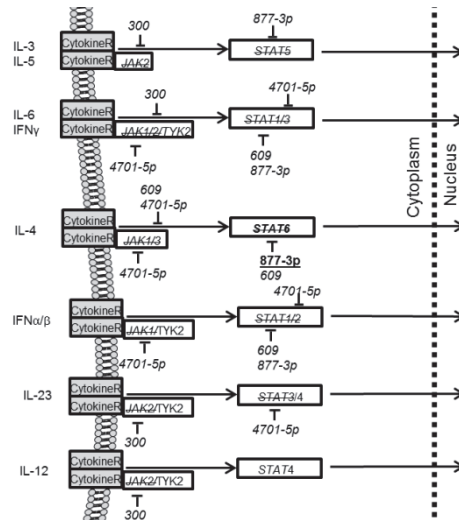

Supplement: Additional file 7: Figure S4. — MAP-kinase and JAK-STAT signaling components are predicted targets of SjS-associated miRNAs. Visualization of predicted miRNA targeting of the MAPK (A) and JAK-STAT (B) signaling pathways. Specific mRNA-miRNA interaction results were based on direct evidence obtained from DIANA Tarbase V.6 database (underlined) and from multiple target prediction programs. (PDF 83 kb) [file 13075_2016_987_MOESM7_ESM.pdf]

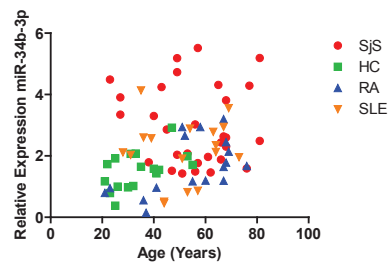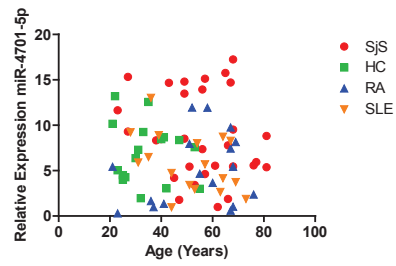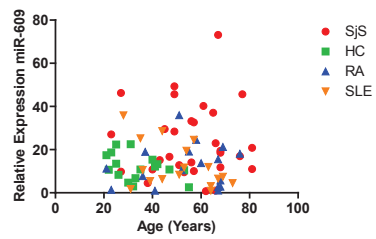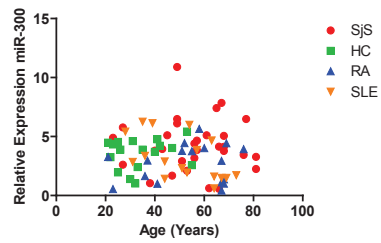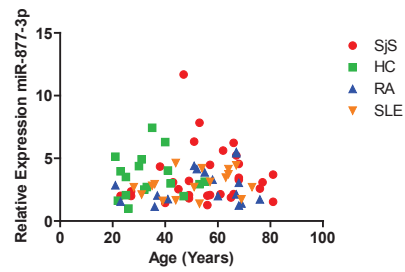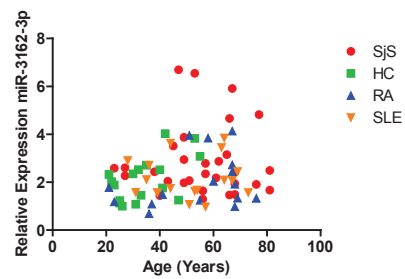

Supplement: Additional file 8: Figure S5. — Analysis of association of age with miRNA expression level indicates no significant differences between HC, SjS, SLE, and RA groups. Based on linear regression analyses, the differences between slopes were not significant among HC, SjS, SLE, and RA groups for miR-34b-3p (P = 0.1974), miR-4701-5p (P = 0.3065), miR-609 (P = 0.511), miR-300 (P = 0.1904), miR-3162-3p (P = 0.4748), and miR-877-3p (0.9712). In addition, although HCs tended to be younger than autoimmune patients on average, they are within the age range of autoimmune patients. (PDF 68 kb) [file 13075_2016_987_MOESM8_ESM.pdf]
