# Supplementary material for: Sjögren’s syndrome-associated microRNAs in CD14+ monocytes unveils targeted TGFβ signaling
Source: Arthritis Res Ther. 2016 May 3;18:95. doi: 10.1186/s13075-016-0987-0 (PMC4855899; doi:10.1186/s13075-016-0987-0)
Supplement: Additional file 5: Table S3. — Database analysis for miRNA target prediction summary. †Databases contain direct miRNA-mRNA target interaction information. (DOC 32 kb) [file 13075_2016_987_MOESM5_ESM.doc]

**Table S3** Database analysis for miRNA target prediction summary.

| MiRNA | Chr. | Tarbase† | miRSystem† | Rna22 | TargetScan | miRNA.org |
| --- | --- | --- | --- | --- | --- | --- |
| miR-34b-3p | 11 | YES | YES | YES | YES | YES |
| miR-609 | 10 | YES | YES | YES | YES | YES |
| miR-3162-3p | 11 | NO | NO | YES | YES | NO |
| miR-300 | 14 | NO | YES | YES | YES | YES |
| miR-4701-5p/miR-588 | 12/6 | NO/YES | NO/YES | YES/YES | YES/YES | NO/YES |
| miR-877-3p | 6 | YES | NO | YES | NO | YES |

†Databases contain direct miRNA-mRNA target interaction information.
